# Preclinical assessment of comfort and secure fit of thermobrachytherapy surface applicator (TBSA) on volunteer subjects

**DOI:** 10.1120/jacmp.v13i5.3845

**Published:** 2012-09-06

**Authors:** Kavitha Arunachalam, Oana I. Craciunescu, Edward J. Markewitz, Paolo F. Maccarini, Jaime L. Schlorff, Paul R. Stauffer

**Affiliations:** ^1^ Department of Engineering Design Indian Institute of Technology Madras Tamil Nadu India; ^2^ Department of Radiation Oncology Duke University Durham North Carolina USA; ^3^ Bionix Development Corporation Paoli Pennsylvania USA

**Keywords:** hyperthermia, conformal microwave array, brachytherapy, chest wall recurrence, breast cancer

## Abstract

A thermobrachytherapy surface applicator (TBSA) was developed for simultaneous heat and brachytherapy treatment of chest wall (CW) recurrence of breast cancer. The ability to comfortably secure the applicator over the upper torso relative to the CW target throughout treatment is assessed on volunteers. Male and postmastectomy female volunteers were enrolled to evaluate applicator secure fit to CW. Female subjects with intact breast were also enrolled to assess the ability to treat challenging cases. Magnetic resonance (MR) images of volunteers wearing a TBSA over the upper torso were acquired once every 15 minutes for 90 minutes. Applicator displacement over this time period required for treatment preplanning and delivery was assessed using MR visible markers. Applicator comfort and tolerability were assessed using a questionnaire. Probability estimates of applicator displacements were used to investigate dosimetric impact for the worst‐case variation in radiation source‐to‐skin distance for 5 and 10 mm deep targets spread 17×13 cm on a torso phantom. Average and median displacements along lateral and radial directions were less than 1.2 mm over 90 minutes for all volunteers. Maximum lateral and radial displacements were measured to be less than 1 and 1.5 mm, respectively, for all CW volunteers and less than 2 mm for intact breast volunteers, excluding outliers. No complaint of pain or discomfort was reported. Phantom treatment planning for the maximum displacement of 2 mm indicated <10% increase in skin dose with <5% loss of homogeneity index (HI) for ‐2 mm uniform HDR source displacement. For +2 mm uniform displacement, skin dose decreased and HI increased by 20%. The volunteer study demonstrated that such large and uniform displacements should be rare for CW subjects, and the measured variation is expected to be low for multifraction conformal brachytherapy treatment.

PACS numbers: 41.20.Jb, 41.75.‐i, 44.

## I. INTRODUCTION

Randomized phase III clinical trials of superficial tumors extending less than 3 cm in diameter have indicated improved complete response (CR) rates when treated with combined heat and radiation compared to radiation treatment alone.^1^, ^4^ Based on clinical literature, tumor thermal enhancement ratio (TER) has been calculated to be 1.5–1.6 for sequential thermoradiotherapy of advanced and recurrent breast cancer, melanomas, and neck nodes.^(5)^ Preclinical data have demonstrated that tumor TER can be increased from 1.5 for sequential treatments to as high as 5.0 when hyperthermia is combined simultaneously with radiation.^(6)^ The benefits of heat‐induced radiation effects on cancer and accessibility of superficial tissue to external radiation and hyperthermia make concurrent thermoradiotherapy an appropriate treatment modality for CW recurrence of breast cancer. Clinical feasibility of simultaneous thermoradiotherapy for CW recurrence reported by Myerson et al.^(7)^ provided motivation to develop hyperthermia applicators for simultaneous thermoradiotherapy of large area superficial disease.^8^, ^12^

**Figure 1:**
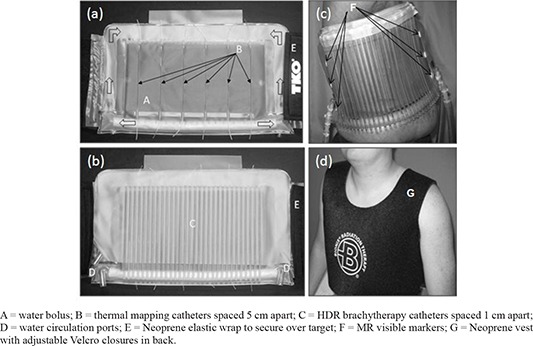
Rectangular shaped TBSA used in the volunteer study: (a) water bolus with thermal mapping catheters on skin contacting side; (b) HDR brachytherapy catheters on the outer side of TBSA; (c) TBSA with MR markers and contrast filled brachytherapy catheters wrapped around a mastectomy volunteer; (d) volunteer with Neoprene over‐garment secured over the TBSA.

A thermobrachytherapy surface applicator (TBSA) has been developed for simultaneous microwave hyperthermia and high dose rate (HDR) brachytherapy treatment of CW recurrence of breast cancer extending 10–15 mm deep from skin surface.^11^, ^13^, ^14^ Figure 1 shows the rectangular shaped TBSA that accommodates a 915 MHz conformal microwave array (CMA) heat applicator with 18 antennas (3×6 array) and $450\;{\text{cm}}^{2}$ treatment area. Superficial hyperthermia is delivered using the CMA through a thin layer of body‐conforming water bolus.^15^, ^19^ A pilot study of using CMA applicator for patients with CW recurrence provided excellent controlled heating and good patient tolerance.^(24)^ A parallel array of HDR brachytherapy catheters on the back side of the water bolus is used to deliver conformal radiation dose to the chest wall disease through the microwave array made of thin copper sheet and coupling water layer.^(20)^ Phantom studies of TBSA reported nearly uniform surface cooling capability of the water bolus and very good applicator conformity to a contoured torso phantom.^20^, ^21^, ^22^ Excellent agreement was obtained between skin dose calculated with the HDR treatment planning system and measured with dosimeters on a tissue equivalent CT phantom.^(23)^

The purpose of this study is to assess the ability to maintain the relative distance between the HDR brachytherapy catheters and CW separated by a body conforming water bolus to deliver the prescribed radiation dose, and to evaluate applicator comfort during a typical thermobrachy therapy treatment. Thus, a preclinical study was carried out on volunteers to assess the comfort and secure fit of TBSA over a predefined region of the upper torso. The specific objectives of the volunteer study are: 1) assess ease of placement and time required to treatment preparations; 2) determine patient comfort limitations; 3) quantify lateral movement of TBSA relative to the predefined target; 4) quantify variation in the HDR brachytherapy catheter (source) to skin surface distance; and 5) determine dosimetric impact of the variations determined in step 4.

## II. MATERIALS AND METHODS

### A. Study protocol

Eligible healthy male and nonpregnant female volunteers, as well as breast cancer patient volunteers with single or double mastectomy, were enrolled on an institute review board (IRB)‐approved protocol. Though the intended disease was CW recurrence of breast cancer, female subjects with intact breast were also enrolled to evaluate applicator secure fit for the most challenging cases. Eligibility criteria included 18 years of age or older with the ability to sign consent and the ability to undergo 90 minutes of magnetic resonance imaging (MRI).

During the study, TBSA with degassed water bolus was wrapped securely around the upper torso using elastic support straps and an external elastic Neoprene vest. Since there is no therapeutic intent, the copper‐based conformal antenna array and radioactive Ir‐192 HDR source were not used in the study. Instead, the 1 cm spaced array of 19 cm long HDR brachytherapy catheters (31 in total) were filled with liquid MR contrast agent (Gadolinium in de‐ionized water) to capture the brachytherapy catheters in the MRI scan. MR‐visible adhesive markers (MM3005; IZI Medical Products, Owings Mills, MD) fixed on the skin and inner surface of TBSA were distributed along the applicator periphery in three rows with two pairs per row to measure applicator lateral movement with respect to the CW (target).

### B. Applicator lateral displacement from CW

Lateral displacement of TBSA with respect to CW was calculated by measuring the distance on each MR image, using the following:

(1) $$d_{c}^{i}=\sqrt{{\left(x_{c,i}^{s}‐x_{c,i}^{a}\right)}^{2}+{\left(y_{c,i}^{s}‐y_{c,i}^{a}\right)}^{2}}$$

where $\left(x_{c,i}^{s},y_{c,i}^{s},z_{i}\right)$ and, expression $\left(x_{c,i}^{a},y_{c,i}^{a},z_{i}\right)$ denotes the center of the corresponding $i^{\text{th}}$ marker is not visible. Distance between marker pairs, calculated by

(2) $$d_{c}={\left\{d_{c}^{i}\right\}}_{i=1}^{N}$$

was calculated for all visible marker pairs in the MR images acquired once every 15 minutes. Distances measured in the 1st scan were used as the baseline measurement to calculate lateral displacement with respect to the CW target.

### C. HDR source‐to‐skin displacement on volunteers

HDR source to skin distance was calculated for each brachytherapy channel (*i*) by computing the radial distance

(3) $$\rho_{i,j}={\left\|\rho_{i,j}^{b}‐\rho_{i,j}^{s}\right\|}^{2}$$

between the center of the contrast‐filled brachytherapy catheter ($\rho_{i,j}^{b}$) and skin surface ($\rho_{i,j}^{s}$), both measured with respect to the MR origin (0, 0, $z_{j}$) of the $j^{th}$ axial image. Radial distances were measured in three axial imaging planes (j=1,2,3) which, coincided with the middle plane of each row of the 3×6 CMA with six antennas per row. Measurements recorded for the same set of M brachytherapy catheters in each imaging plane for the subsequent MR scans were used to track HDR source displacement from the CW. Deviation in the radial distance computed with respect to the first MR scan $\rho^{\left(0\right)}$ was used to quantify HDR catheter displacement from CW target

(4) $$\Delta \rho (k)=\left|\rho (0)‐\rho (t_{k})\right|$$

for k=15, 30, 45, 60, 75, and 90. The large number of measurements (3M) per scan allowed computation of density distribution and confidence interval for applicator displacement for each volunteer.

### D. Dosimetric impact of HDR source‐to‐skin displacement on phantom

Probabilistic distribution curves estimated from the radial applicator displacement in Section C above were used to assess the dosimetric impact of radial applicator displacement from CW on a homogeneously layered elliptic phantom (32×20×60 cm) invariant along the z‐axis (60 cm). To account for the thickness of the copper heating array, a CMA applicator was used with the TBSA in the phantom study. Due to the significantly low attenuation of photon beam by the thin copper CMA and subsequent absorption of the secondary electron emission by the bolus reported in Taschereau et al. and Craciunescu et al.,^14^, ^23^ dose calculations were carried out using BrachyVision (Varian, Palo Alto, CA). Initial plans were generated using CT scans of the elliptic phantom with 8 mm bolus to deliver 100 cGy to cover a patient treatment volume (PTV) defined at 5 mm and 10 mm depths spread 17×13 cm across the phantom surface. Dwell times of the HDR source were optimized for 1 cm spacing between the adjacent HDR catheters.

Subsequently, plans were generated for uniform variation in bolus thickness (thinning or thickening) based on the density distribution curves estimated in Section C above. Dwell times were kept the same and dose renormalization was not done, in order to assess the dosimetric impact of applicator displacement from the CW. Standard dose parameters, namely coverage index V(x) or percentage of target volume receiving at least x% of dose (where x=100, 130, 140), dose received by 100% of the target D100, homogeneity index (HI=V100–V140)******, skin dose (cGy), and skin gradient (cGy/mm) were calculated and compared between an unperturbed (8 mm constant thickness bolus) and the perturbed configurations (uniformly thicker and thinner bolus with respect to 8 mm bolus).

### E. Comfort assessment

The ease of TBSA placement and time required to complete volunteer preparations for heat/ HDR brachytherapy treatment were documented for each subject. A questionnaire filled out by the volunteers at the end of the study was used to assess TBSA comfort and tolerance during the 90 minute period, which corresponds to 150% of the typical hyperthermia treatment time. A numeric scoring sheet was used to convert the qualitative metrics of comfort experienced by the volunteers.

## III. RESULTS

### A. Study population and applicator conformity

**Figure 2:**
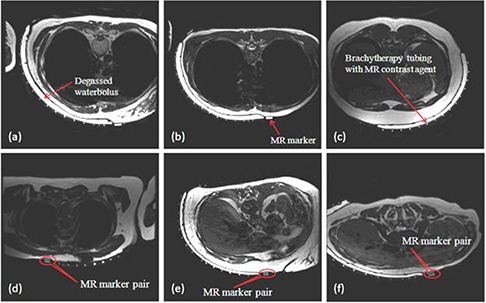
MR images of volunteer CW: (a)–(c) axial images of mastectomy and healthy volunteers with excellent conformity of TBSA to CW; MR images indicate MR visible marker pairs on the skin and TBSA that were used to assess applicator lateral displacement. The bright dots above the water bolus from the liquid MR contrast filled inside the parallel array of brachytherapy catheter channels were used to assess radial displacement of HDR catheters from the CW surface.

Table 1 lists the subjects enrolled in the volunteer study. Figure 1 shows the typical placement of the rectangular TBSA on a volunteer. The applicator was positioned with considerable ease and the average time to prepare the subject for scanning was 15 minutes. MR images of volunteers in Fig. 2 show excellent applicator conformity to the CW. MR markers with bright signal intensity appearing in all imaging planes were used for analysis.

**Table 1:** Volunteers enrolled in the preclinical volunteer study.

*Volunteer*	*Gender*	*Age*	*Remarks*	*Imaging Region*
1	Male	25	None	Chest wall
2	Female	40	Single mastectomy	Chest wall
3	Male	24	None	Chest wall
4	Female	24	Healthy, intact breast	Intact breast
5	Female	30	Double mastectomy	Chest wall
6	Female	30	Intact breast, lumpectomy	Intact breast
7	Female	56	Single mastectomy	Chest wall
8	Female	57	Single mastectomy	Chest wall

### B. Applicator lateral displacement on volunteers

**Figure 3:**
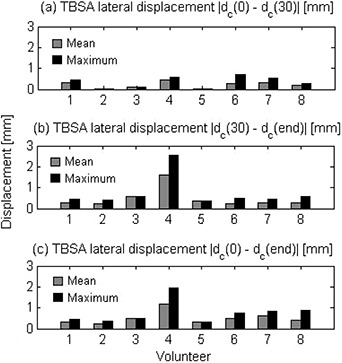
Lateral displacement of applicator along the CW: (a) applicator displacement 30 minutes after the baseline scan (i.e., displacement at start of thermobrachytherapy treatment); (b) displacement at the end of study measured with reference to the data obtained 30 minutes after the baseline (i.e., displacement during treatment); (c) overall displacement measured between the first (planning) and last (end of treatment) scans.

(Figure 3a) shows the average and maximum values of the lateral applicator displacements $\left(|d_{c}(0)-d_{c}(30)|\right)$ measured in this study. These measurements correspond to the lateral displacement that can be expected at the start of thermobrachytherapy (i.e., 30 minutes after the baseline scan is obtained for HDR brachytherapy planning). (Figure 3b) gives the displacement $\left(|d_{c}(30)-d_{c}(end)|\right)$ that can be expected during a 60 minute thermobrachytherapy treatment. (Figure 3c) shows lateral displacement $\left|d_{c}(0)-d_{c}(end)\right|$, at the end of study measured with respect to the baseline scan. Figure 3 demonstrates that the TBSA remained in position throughout the study with less than 1 mm lateral movement from the skin surface for the CW volunteers (male & postmastectomy subjects) compared to the volunteer group with intact breast (subjects 4 and 6).

### C. HDR source‐to‐skin displacement on volunteers

**Figure 4:**
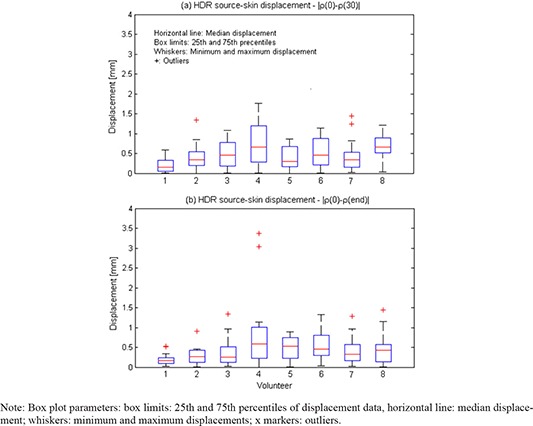
Radial displacements of HDR brachytherapy catheters from the CW surface: (a) applicator displacement 30 minutes after baseline scan (i.e., displacement at start of thermobrachytherapy treatment); (b) displacement measured between the first and last scans.

**Figure 5:**
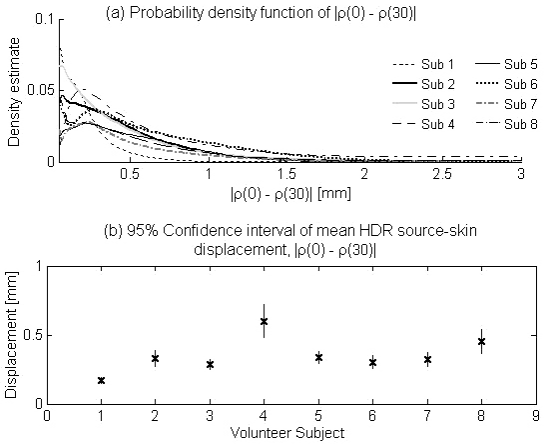
Density distribution curves (a) of the 3M HDR catheter‐CW displacement measurements |ρ(0)−ρ(30)| which correspond to the relative shift in position of TBSA catheters at the start of thermobrachytherapy treatment, 30 minutes after the baseline data was obtained for HDR pretreatment planning. 95% confidence interval (b) (solid line) for the mean (cross marking) displacement of |ρ(0)−ρ(30)|. Density distributions and the estimated confidence intervals indicate very small variation in radial applicator displacement.

The number of brachytherapy channels (M) used to measure HDR source‐to‐skin displacement ranged from 16 to 30, depending on catheter visibility in the three imaging planes (mean=22, median=21). (Figure 4a) shows the statistics computed using the 3M radial applicator displacement data (|ρ(0)−ρ(30)|) 30 minutes after the start of the study. (Figure 4b) shows the displacement measured at the end of study with respect to the baseline scan. The median displacement indicated by horizontal line inside the box plot is less than 1 mm for both volunteer groups (CW, intact breast) and time instants (30, 90 minutes). It can be observed that the span of the radial displacement data is larger for intact breast volunteers compared to the CW group. Similar observations were made for |ρ(30)−ρ(end)| (data not shown).

(Figure 5a) shows the probability distribution function estimated for the radial applicator displacement, |ρ(0)−ρ(30)| for the individual volunteer. The skewed distribution curves observed for all volunteers indicate a low variation in the applicator displacement from CW. It should also be noted that the outliers, which correspond to the tail end of the distribution, occur beyond 2 mm. The 95% confidence interval for the mean value of the radial displacement (|ρ(0)−ρ(30)|) in (Fig. 5b) indicates a tighter span (≤ 5 mm) about the mean for all volunteers. Similar performance was observed for |ρ(30)−ρ(end)| and |ρ(0)−ρ(end)| (data not shown).

### D. Dosimetric impact of HDR source‐to‐skin displacement on phantom

**Figure 6:**
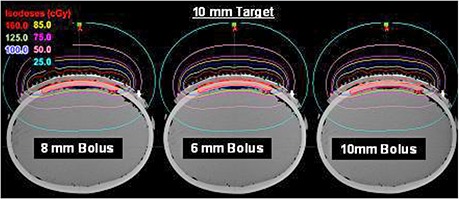
Transverse isodose distributions for a prescription of 100 cGy to 10 mm deep target in the case of undisturbed bolus (8 mm) and bolus thickness variation of ±2 mm.

**Figure 7:**
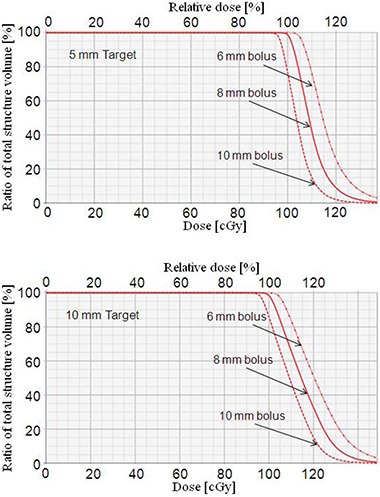
Dose volume histograms for 5 mm (top) and 10 mm (bottom) deep targets calculated for the nominal 8 mm thick water bolus (solid lines) and for thicker (10 mm, dashed lines) and thinner (6 mm, dotted‐dashed lines) water bolus.

A 2 mm displacement observed at the tail end of the distribution functions in (Fig. 5a) was used to compare the dosimetric computed for uniform perturbation in bolus thickness from the average thickness (8 mm). Figure 6 shows the transverse isodose lines for a prescription of 100 cGy to 10 mm deep target in case of unperturbed water bolus (8 mm) and bolus thickness variation of ±2 mm. Figure 7 shows the dose volume histogram (DVH) for two targets when displaced by ±2 mm uniformly across the treatment planning surface. The dose metrics derived from Figs. 6 and 7 are summarized in Table 2. This table also includes skin dose and skin dose gradient for ±1 mm displacement, as we determined that the median as well as the mean HDR source‐to‐skin displacement is less than 1 mm (see Figs. 4 and (5b). Notably though, the V(x), D100 and HI metrics were not calculated for ±1 mm, as the treatment planning software lacked the ability to distinguish contours down to one mm resolution.

**Table 2:** Summary of dose metrics for 5 mm and 10 mm deep targets and ± 2 mm variation in bolus thickness.

	*Target 5 mm*			*Target 10 mm*		
*Dose Metrics*	*0 mm*	−1/−2 mm	+1/+2 mm	*0 mm*	−1/−2 mm	+1/+2 mm
Skin dose [cGy]	123.4	128.7/134.0	119.2/115.5	132.0	136.7/140.8	128.5/125.0
Skin gradient [cGy/mm]	4.2	4.2/5.3	4.2/3.6	3.5	3.5/4.1	3.5/3.2
HI (V100–V140) [%]	98.4	97.9	76.8	96.0	91.9	85.3
V(100) [%]	98.7	100.0	76.8	98.3	100.0	85.7
V(130) [%]	1.9	7.3	0.22	9.8	23.8	2.6
V(140) [%]	0.3	2.11	0.0	2.3	8.1	0.4
D(100) [cGy]	98.4	101.1	93.9	96.4	99.2	93.4

Note: Coverage index, V(x) is the percentage of target volume receiving x% of dose or more (x=100,130,140), HI is the homogeneity index and D(100) is the dose received by 100% of target. V(x), D(100) and HI were not calculated for ± 1 mm displacement as the treatment planning software lacks this resolution in defining planning target volumes.

For negative displacements, the most relevant metric to look at is skin dose. The data in Table 2 infer that differences in skin dose are <5% for 1 mm and <10% for 2 mm displacements with only modest change in skin gradient. The V(100) and D100 improve with a negative displacement, and changes in the hot spots are minimal but, as expected, are larger for the deeper target. For positive displacement, meaning the water bolus becomes thicker by 1–2 mm, skin dose is no longer an overdose concern, since it decreases. The dosimetric metrics of importance are the coverage indexes. With a 2 mm uniform increase in water bolus thickness across the treatment surface, the HI varies by 20%, stemming from the large variation in V100.

### E. Applicator comfort assessment by volunteers

**Figure 8:**
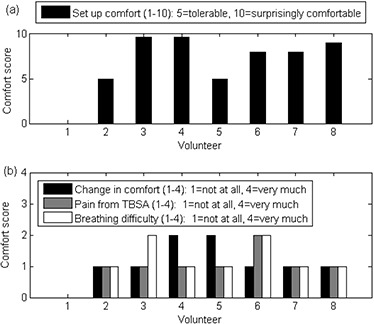
Numerical scores recorded by the volunteers on the comfort level of TBSA during the preclinical study. Note that the applicator and procedure were well‐tolerated (score ≥ 5 in and comfort remained almost the same (score ≤ 2 in during the study. No pain, breathing difficulty or discomfort was reported by any of the volunteers.

Figure 8 summarizes the comfort questionnaire filled out by volunteers at the end of the study. (Numerical scores of applicator comfort were not available for the 1st volunteer as the questionnaire was under revision. However, oral feedback from this healthy male volunteer indicated comfortable applicator placement.) The applicator setup was comfortable and well‐tolerated by all volunteers, and the comfort level did not change much during the 90 minute study. The comfort questionnaire revealed a small number of minor complaints such as feeling a contact point under the arm or over the shoulder. No complaints of pain, breathing difficulty or significant discomfort were recorded by any of the subjects.

## IV. DISCUSSION

This effort describes the testing of the comfort and secure fit of a thermobrachytherapy surface applicator developed for simultaneous heat and HDR brachytherapy treatment of CW recurrence of breast cancer in postmastectomy patients with laterally diffuse disease extending < 15 mm from the skin surface.^20^, ^21^, ^23^, ^24^ The advantages of HDR brachytherapy include:^(25)^ accuracy and precision of tumor specific radiation dose delivery, fewer side effects, excellent coverage of irregularly shaped target disease, and a shorter course of treatments compared to standard fractionated external beam radiotherapy. The safe use of customized surface molds for HDR brachytherapy treatment of superficial disease has been reported for chest wall recurrence in pre‐irradiated postmastectomy patients,^(26)^ skin carcinoma,^(27)^ cancers of the scalp,^28^, ^29^ head and neck cancer,^(30)^ and scar boost radiation after breast reconstruction.^(31)^ The body conforming water bolus of the TBSA with a thin layer of uniform thickness filter foam acts as a surface mold for HDR brachytherapy. During HDR brachytherapy, it is vital to maintain the preplanned distance between the brachytherapy catheters and CW surface. This volunteer study was initiated to measure the spatial variation in the distance between HDR brachytherapy catheters and CW surface over a 90 minute period. The 90 minute study duration includes 30 minutes for initial scanning and HDR pretreatment planning, and 60 minutes for thermobrachytherapy treatment. Study was conducted in the absence of the copper heating antenna array and HDR source to assess applicator comfort and secure fit. Another reason for not including the copper heating array was because of the electromagnetic (EM) interference provided by the CMA applicator to the MR pickup coils.

Excellent applicator conformity to the torso was observed for all CW volunteers (Fig. 2), which is the intended tissue target for TBSA. For the chest wall volunteers, the sequential scans demonstrated less than 1.0 mm maximum (0.78 mm median) movement laterally across the target surface. The maximum lateral displacement was measured as 2.7 and 0.75 mm for 4th and 6th intact breast volunteers, respectively (Fig. 3). Excluding the outlier measurements reduced the maximum lateral displacement to less than 2 mm for the 4th volunteer. The mean lateral applicator displacement was well below 1 mm for both intact breast volunteers, excluding the outlier measurement of 4th subject. Dosimetric impact of this 1 mm mean lateral applicator displacement from CW target was not simulated since a 1 mm change in target contour could be resolved by the treatment planning software.

The maximum HDR source‐to‐skin displacement was measured to be less than 1.5 mm for the CW volunteers (Fig. 4). The maximum radial displacements measured for the intact breast group were 3.35 and 1.3 mm for volunteer #4 and #6, respectively (Fig. 4). The largest displacement of 3.35 mm noted for the 4th volunteer is a measurement outlier, as seen in (Fig. 4b). Exclusion of the outlier measurements yielded less than 2 mm variation in HDR source‐to‐skin displacement for the intact breast volunteer group. The mean and median radial displacements computed for the intact breast group was less than 1 mm (see Figs. 4 and 5) similar to the CW group. The larger variation in applicator displacement measured for the intact breast group compared to the CW group is due to practical difficulties in fitting the applicator to the intact breast. Displacement measurements in Figs. 3 to 5 obtained from the sequential MR images indicate that the TBSA can be secured to the CW with low variation (< 2 mm) during an hour long thermobrachytherapy of the CW disease.

Dosimetrics summarized in Table 2 for an unperturbed (8 mm bolus) and perturbed treatment setups (10 and 6 mm bolus) calculated for ±2 mm variation based on data distribution in (Fig. 5a) indicated about 10% increase in skin overdose for the 10 mm deep target with less than 3% variation in HI. However, it should be noted that the skin is also a part of the CW disease and variation in skin dose is less than 10% for a uniform decrease in bolus thickness across the treatment surface. For positive displacement, the variation in HI index is relatively larger (as much as 20%) due to the increase in the depth of the target location from the HDR source. Dose delivered to the skin is relatively lower for positive displacement due to radiation attenuation inside the thicker bolus. It should be noted that the dosimetric in Table 2 provides the worst‐case scenarios that can result due to change in bolus volume between treatments in a multifractionated thermobrachytherapy treatment. In reality, the bolus volume is maintained the same between and during treatments to avoid perturbations in EM power deposition inside tissue. A detailed brachytherapy treatment planning of this volunteer data is expected to provide better dosimetric than the phantom study and will determine the clinical acceptability of the applicator displacement measurements.

The overall comfort rating of the setup varied between tolerable and surprisingly comfortable for the postmastectomy volunteers, as seen in Fig. 8. Comfort level remained essentially the same for the duration of study for all volunteers, and none experienced pain or breathing difficulty. Based on prior clinical experience with the CMA heat applicator,^(24)^ sweating and minor discomfort from the hyperthermia treatment are expected to remain the most significant factors in patient comfort during thermobrachytherapy. That expectation was confirmed in the results of this study. In clinical use of the TBSA, the anticipated causes for applicator movement relative to chest wall target will consist of patient movements on and off the CT table for radiation planning scans, patient transfer to the hyperthermia/brachytherapy delivery bed, and minor patient movements during the 60 min treatment interval. As tested in this study, such applicator movements could be minimized during treatment using MR visible marker pairs on skin and applicator, and securing the TBSA with elastic straps and an overlying elastic vest.

## V. CONCLUSIONS

A quantitative assessment of the comfort and secure fit of a thermobrachytherapy surface applicator (TBSA) was carried out on volunteer subjects for 90 min to simulate a complete procedure involving pretreatment scans followed by time sufficient for planning and delivery of simultaneous heat and radiation to a chest wall target. Applicator displacement relative to the chest wall target was assessed with MR scans of the torso of each volunteer at 15 min intervals for 90 min, with realistic patient movement allowed between scans. As expected, higher displacement was measured for volunteers with intact breast compared to those with chest wall target. This preclinical study confirms that simultaneous thermobrachytherapy is feasible with an easy‐to‐use TBSA that provides uniform treatment of large area CW disease while remaining comfortable for the patient. The outcome of this volunteer study strongly supports future clinical testing of TBSA as a means to enhance the synergism of heat and radiation in diffuse superficial disease.

## ACKNOWLEDGMENTS

This research was supported by NIH grants RO1‐CA70761, P01‐CA‐042745, and R44‐CA104061 with Bionix Development Corporation. The authors would like to thank hyperthermia treatment technologist Wendy Covington, and MR technician Kevin Kelly of the Department of Radiation Oncology, Duke University Medical Center, for their assistance with the volunteer study.

## Supporting information

Supplementary Material Files

Supplementary Material Files
