# Supplementary figures and images for: Preclinical assessment of comfort and secure fit of thermobrachytherapy surface applicator (TBSA) on volunteer subjects
Source: J Appl Clin Med Phys. 2012 Sep 6;13(5):223–35. doi: 10.1120/jacmp.v13i5.3845 (PMC3439213; doi:10.1120/jacmp.v13i5.3845)

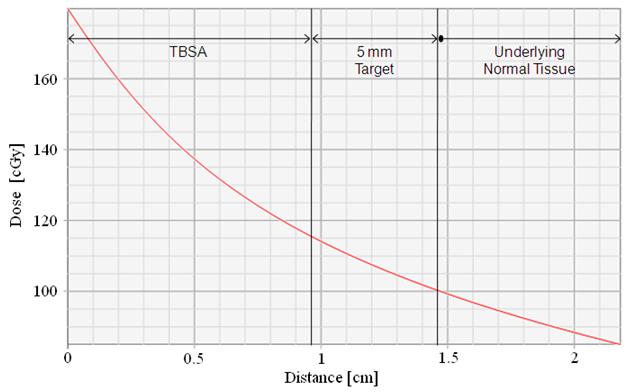

Supplement: Supplementary file 1 — Supplementary Material Files [file ACM2-13-223-s001.jpg]

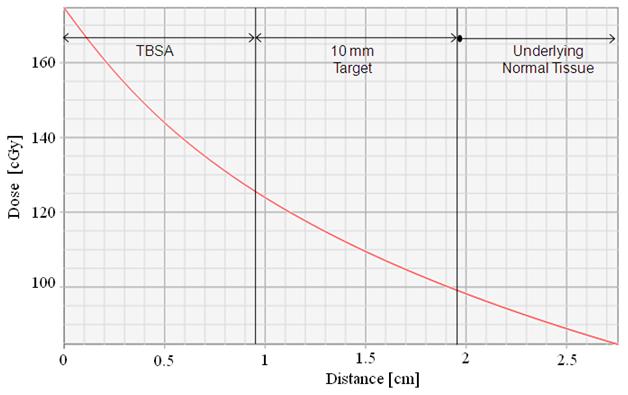

Supplement: Supplementary file 2 — Supplementary Material Files [file ACM2-13-223-s002.jpg]
